# Immediate endoscopic management of complete iatrogenic anterior urethral injuries: A case series with long-term results

**DOI:** 10.1186/1471-2490-5-13

**Published:** 2005-11-09

**Authors:** Pankaj N Maheshwari, Hemendra N Shah

**Affiliations:** 1 Department of Urology, R. G. Stone Urological Research Institute, Mumbai, India; 2 Presently consultant urologist, Bombay Hospital, Indore, India

## Abstract

**Background:**

Urethral injury produces partial or complete disruption of the urethral integrity. Advances in endourology have made endoscopic management of most of these injuries feasible without greatly compromising the final result. We report our institutional experience of immediate endoscopic realignment of complete iatrogenic anterior urethral injury.

**Methods:**

From May 1997 to May 2003, seven patients with complete anterior urethral disruption were managed by immediate endoscopy guided splinting of urethra. Retrograde urethroscopy, combined with fluoroscopic guidance and in some cases antegrade cystoscopy through a suprapubic stab cystostomy was performed. A guide wire was negotiated across the disruption. Later, a 16 F Foley catheter was placed for 1–3 weeks. Patients were followed up at 1, 3, 6 and 12 months and then yearly to assess the long-term outcome of endoscopic management.

**Results:**

Immediate endoscopic realignment was achieved in all patients. Three patients developed recurrence at six months; that was treated by optical urethrotomy. Only one patient developed multiple recurrences over an average follow-up of 49.2 months (range 7 to 74 months). He was offered open end-to-end urethroplasty at twenty months after third recurrence. Thus immediate endoscopic realignment avoided any further intervention in four patients (57.14%); while after an additional optical urethrotomy, urethroplasty could be avoided in six patients (87.2%).

**Conclusion:**

Immediate endoscopic realignment of traumatic urethral disruption is a feasible, safe and effective treatment modality for management of patients with iatrogenic complete anterior urethral injuries.

## Background

Anterior urethral rupture is rare and is usually iatrogenic, during catheterisation or urethral dilatation. Trauma resulting from inflating a Foley balloon in the urethra occurs frequently. It may also result from blunt trauma e.g. saddle injury.

The definitive management of anterior urethral injury remains controversial [1]. Initial supra pubic cystostomy and delayed urethral re-construction had been considered as a reference standard for managing posterior urethral injuries [2]. This approach has problems like need of a supra pubic drainage for prolonged period (6 weeks to 3 months) as well as formation of an inevitable urethral stricture requiring reconstructive urethroplasty.

Recent advances in endourological techniques have made primary realignment feasible to perform with minimal manipulation. This technique realigns the urethra over a urethral catheter that splints the urethra till the healing occurs [3]. We herewith retrospectively analyse our experience with immediate endoscopic realignment of iatrogenic complete anterior urethral injuries.

## Methods

### Patients

From May 1997 to May 2003, seven men were diagnosed at our institute with complete iatrogenic anterior urethral injury. Patients had acute retention of urine and/or bleeding per urethra and were treated on emergency basis. A detailed clinical evaluation was performed and previous treatment records were reviewed to know the exact pathology in the urethra. Patients were subjected to primary endoscopic realignment of their urethral injury. An informed consent was taken for supra pubic cystostomy in all cases.

### Operative technique

Under spinal or epidural anaesthesia patients were placed in lithotomy position with the legs on adjustable leg supports. A dynamic ascending urethrogram was performed under fluoroscopic guidance to confirm extravasation of contrast. Complete rupture was suspected when there was failure to delinate proximal urethra during urethrography.

Initial urethroscopy was done with 17 F cystoscopic sheath and a 0° telescope. Normal saline irrigation was kept to as minimum as possible. Clots in the urethra were gently evacuated to reach the site of urethral disruption. Once complete lack of urethral mucosal integrity was confirmed endoscopically following steps were used to delineate proximal urethra:

#### Step 1- Retrograde endoscopic delineation of proximal urethra

Bladder was punctured suprapubically with 16 F initial puncture needle and methylene blue was injected in bladder. A suprapubic pressure was applied with the simultaneous urethroscopy to identify possible efflux of methylene blue at the site of trauma. An attempt was made to gently negotiate 0.035"hydrophilic glide wire through the area effluxing methylene blue. If attempt succeeded, position of glide wire in bladder was confirmed fluoroscopically. A stiff zebra wire replaced the glide wire. This served as a guide for subsequent urethroscopy.

#### Step 2- Antegrade fluoroscopic guided delineation of proximal urethra

If the glide wire could not be negotiated retrogradely; an angled tip glide wire was passed from initial puncture needle into the bladder. Under fluoroscopic guidance multiple attempts were made to negotiate glide wire into posterior urethra through bladder neck. Once glide wire reached the posterior urethra a 6 F open-ended ureteric catheter was passed over it into posterior urethra & a glide wire was exchanged for a guide wire.

#### Step 3- Antegrade endoscopic delineation of proximal urethra

If fluoroscopically guided attempts to negotiate guide wire in to the posterior urethra failed; it was supplemented with endoscopic guidance. The supra pubic tract was dilated up to 24 F using Alken dilators and an Amplatz sheath was than used as a conduit to pass a rigid cystoscope into bladder. Bladder neck was than explored with antegrade cystoscopy and an open ended ureteric catheter was passed into the posterior urethra.

Simultaneous retrograde urethroscopy was used to explore the site of urethral trauma. The glide wire passed through the suprapubically placed open-ended ureteric catheter was pushed down to the site of urethral trauma. This glide wire was easily identified during the retrograde urethroscopy, grasped with cystoscopic grasping forceps and pulled out through the external meatus. The 6 F open-ended ureteric catheter was introduced percutaneously over the glide wire till it appeared at the meatus. Later the glide wire was exchanged with super stiff zebra wire. This zebra wire then acted as a guide for 16 F Foley catheter, which was placed perurethrally in the bladder. If the supra pubic tract was dilated, an 18 F Foley catheter was placed suprapubically as a safety measure.

#### Postoperative course and follow-up

The suprapubic catheter, if placed was removed on first postoperative day before the patient was discharged from hospital. The urethral catheter was left in place for 1 to 3 weeks, depending on severity of injury. A dynamic urethrogram was performed by the side of catheter to confirm absence of extravasation of contrast before catheter removal. Patients were followed up at 1, 3, 6 and 12-month interval and than yearly. They were monitored with history, physical examination, uroflow rate measurement and ultrasonography guided determination of post void residual urine volume. Development of obstructive urinary symptoms, decrease in flow rate and/or increase in post-void residual urine volume was evaluated by retrograde urethography or Cystoscopy.

## Results

From May 1997 to May 2003, seven men with age ranging from 36 years to 68 years (mean age 52 years) were diagnosed at our institute with complete iatrogenic anterior urethral injury. Three of these patients had the balloon of a Foley catheter inflated in the bulbar urethra and were referred with catheter in situ draining blood. Three patients were known case of urethral stricture and underwent urethral dilatation followed by bleeding per urethra. One patient had undergone optical urethrotomy for a 1 cm bulbar urethral stricture. However during surgery, glide wire accidentally came out after partial incision of the stricture and later on proximal urethra could not be identified. The patient was later referred to other institute for further management. The detailed demographic data, cause of urethral trauma, primary urethral pathology, duration between injury and endoscopic realignment, operation time, operative procedure required, duration of hospital stay, duration of perurethral catheter & followup course of each patient is mentioned in table 1.

**Table 1:** Patients demographic, surgical & follow-up data.

Sr. No.	Age	Cause of urethral Trauma	Past urethral Pathology	Duration between trauma and surgery (Hrs.)	OR time (min)	Procedure needed for endoscopic alignment	SPC needed	Hospital Stay (hours)	Duration of perurethral catheterization (weeks)	Duration of followup (months)	Recurrence
1	57	Visual internal urethrotomy	Bulbar urethral stricture	8	45	Suprapubic Amplatz	Yes	36	3	74	At 6 month
2	48	Urethral dilatation	Proximal penile stricture	4	15	Retrograde glide	No	14	2	66	Nil
3	68	Catheterization	Acute retention after piles surgery	16	40	Suprapubic Amplatz	Yes	28	1	58	Nil
4	53	Catheterization	Acute urinary retention	4	30	Suprapubic Amplaz	Yes	28	1	56	Nil
5	42	Urethral dilatation	Stricture Urethra	2	35	Suprapubic Amplatz	Yes	36	2	47	At 4 months
6	36	Urethral Dilatation	Stricture Urethra	18	30	Suprapubic needle access	No	18	3	36	At 6, 11 & 20 months followup. Open end to end Urethroplasty done
7	60	Catheterization	Acute retention after bilateral hernia surgery	6	35	Suprapubic Amplatz	Yes	24	1	7	Nil

All patients had complete loss of urethral continuity. Bulbar urethra was affected in four patients and the remaining three patients had proximal penile urethral rupture. Glide wire was negotiated retrogradely only in one patients & the remaining six patients needed antegrade (suprapubic) access. Rigid antegrade cystoscopy was needed through suprapubically placed Amplatz sheath in five patients.

Procedure was successful in all the seven patients. Suprapubic catheter kept in five patients was removed on first post-operative day. Per urethral catheter was kept for one week in three patients, two weeks in two patients and for three weeks in remaining two patients. No patient had extravasation of contrast during dynamic retrograde urethrogram performed before catheter removal. All patients were asymptomatic and had insignificant post-void residue on sonography during 1-month follow-up. The peak uroflow rate was 19.7 ml/sec. (range 15–24 ml/sec) & the post-void residual urine was 38 ml (range 10 to 55 ml) on ultrasonography measurement. By six months of follow-up three patients developed stricture at the site of trauma. Of these two patients had injury during dilatation for the stricture urethra. All these three patients were treated by optical urethrotomy and were taught self-calibration of urethra for an average period of 6 months. With the average follow-up of 49.2 months (range 7–74 months) all, except one patient are asymptomatic with no further recurrence of stricture urethra. Only one patient developed multiple recurrences & was offered open end-to-end urethroplasty at twenty months after third recurrence. Hence four of our patients (57.14%) were disease free during their follow-up after their immediate endoscopic realignment for urethral trauma. After an additional optical urethrotomy, urethroplasty could be avoided in six patients (87.2%).

## Discussion

Controversy continues regarding proper management of traumatic urethral disruption. The suggested surgical treatment modalities include a) immediate primary simple realignment over a stenting catheter b) immediate primary suture repair c) immediate suprapubic cystostomy alone, with delayed elective urethroplasty for the resulting stricture. Many urologists believe that delayed urethral reconstruction is the safest method [2]. However, placement of suprapubic catheter significantly impairs the patient's quality of life.

Advances in endoscopic instrumentation and techniques have expanded our armamentarium for safe and effective treatment of urethral strictures. In a recent review on management of urethral trauma, it is stated that a stenting urethral catheter may be useful in management of anterior urethral injury in some cases [4]. However the exact role of primary endoscopic realignment in management of complete iatrogenic urethral injury was not specified.

Towler JM et al initially described realignment of traumatic urethra over a splinting catheter by instrumentation and endoscopy via both suprapubic and urethral routes in an effort to decrease patient morbidity [5]. Moudouni SM et al described early endoscopic realignment of posterior traumatic urethral disruption [6]. On follow up of 68 months they concluded that the urethral continuity could be established without any increase in the incidence of impotence, stricture formation or incontinence. In case of failure, endoscopic realignment doesn't compromise the results of secondary urethroplasty. In another study Jepson BR et al employed a variety of endourological techniques to achieve urethral continuity in 8 patients [3]. The average time to realignment was 9.5 days. With a mean follow up of 50.4 months four patients required subsequent internal urethrotomies with eventual voiding stabilisation over the course of 11 months. Olapede J et al used either both antegrade & retrograde cystoscopy (with or without fluoroscopy), or flexible retrograde urethroscopy alone for primary endoscopic realignment of posterior urethral trauma [7]. Similar good results were reported in various studies from different institution [8-11]. Thus the technique of immediate endoscopic realignment of urethral injuries is widely employed for management of posterior urethral injuries.

Since the anterior urethral injuries are rare, accepted treatment methods are commonly based on relatively few patients. Benchekroun A et al on reviewing their 23 patients with anterior urethral trauma recommended delayed end to end anastomosis for total rupture, reserving immediate repair in patients with associated penile fracture [12]. Dobrowlski ZF et al in their series of 255 patients with anterior urethral injury found iatrogenic trauma during catheterisation or cystoscopy to be commonest (206 patients) [13]. The treatment was conservative in 193 patients and surgical in 62. Partial rupture of anterior urethra will heal after urinary diversion alone with excellent results [14]. However the most common complication of complete urethral rupture is development of urethral stricture [15]. The goal of immediate primary realignment lies in the ability to perform the procedure in as timely a fashion as possible with minimal disruption to the already traumatized tissue. Realignment of the urethra will produce more anatomically aligned stricture and obviate the need for long-term suprapubic tube drainage [15]. Londergan TA et al employed early fluoroscopic realignment for traumatic urethral injury [9]. Of 2 patients with anterior urethral trauma, the procedure was successful in one patient. The patient later required visual internal urethrotomy at 35-month follow-up. Keeping the results of primary endoscopic realignment of posterior urethral injuries in mind, Ying-Hao S et al were first to employ technique of urethroscopic realignment for treating 16 patients with ruptured bulbar urethra [1]. They felt that since the gap in the anterior urethra after disruption is relatively short and the proximal end of disrupted anterior urethra is more immobile, a combined suprapubic intervention was not required to re-establish anterior urethral continuity. However only 4 patients in their series had complete injury. They found procedure feasible in all these patients with only 1 patient requiring intermittent self-dilatation once weekly. They treated all patients on outpatient basis under local anaesthesia. Based on their results, they recommended primary endoscopic urethral reunion as an ideal, cost effective treatment in treating male patients with bulbar urethral disruption. Nakajima et al employed a thin trocar needle for endoscopic realignment of complete disruption of bulbar urethra resulting from a straddle injury [16]. They confirmed exact suprapubic trocar position with flexible antegrade cystoscopy and placed a guide wire in proximal urethra and found the procedure safe, simple and reproducible. Ku JH et al supplemented above results by comparing immediate versus delayed treatment [15]. Of 95 patients studied, 46 had complete disruption. Primary endoscopic realignment was successful in 65 patients. The remaining patients underwent delayed repair. In their experience, the incidence of urethral stricture after complete disruption was statistically low in patients undergoing immediate realignment as compared with those patients treated with delayed repair (31.3% vs. 68.8%). They suggested that better outcomes could be obtained when immediate urethral realignment is successful in patients with bulbar urethral disruption.

In view of encouraging results of primary endoscopic realignment of posterior urethral disruption, we employed the similar technique for treating our patients with iatrogenic anterior urethral injuries from May 1997 onwards. Our results are encouraging and except for three patients who developed stricture at 6 months follow-up, rest all patients had a patent urethra without any urinary symptoms. We believe that the development of stricture in these three patients was attributable to presence of urethral stricture before urethral trauma rather than the long-term result of immediate endoscopic realignment that they underwent for urethral trauma. Two out of these three patients had documented stricture for which they had undergone dilatation in past. During dilatation, trauma resulted causing urethral disruption. Patients who developed stricture during follow-up were easily managed by optical urethrotomy, avoiding the need for open urethroplasty &/or long-term supra-pubic urinary diversion. Based on our experience we feel that immediate endoscopic realignment of iatrogenic anterior urethral disruption is feasible, safe and effective. The major drawback of our study is small number of patients and lack of comparison with patients who were managed by delayed method. A prospective randomised trial with larger number of patient is necessary to evaluate definitive role of immediate endoscopic realignment in management of complete anterior urethral injuries.

## Conclusion

Immediate endoscopic realignment of complete iatrogenic urethral disruption is a feasible, safe and effective treatment modality for management of these patients.

## Competing interests

The author(s) declare that they have no competing interests.

## Authors' contributions

HS participated in the design of the study, performed the review of records, data acquisition, statistical analysis and helped to draft the manuscript. PM revised the manuscript critically for important intellectual content and finally approved the manuscript. Both authors read and approved the final manuscript.

## Pre-publication history

The pre-publication history for this paper can be accessed here:


